# Knowledge, perceptions, and practices of axial spondyloarthritis diagnosis and management among healthcare professionals: an online cross-sectional survey

**DOI:** 10.1007/s00296-024-05638-w

**Published:** 2024-06-22

**Authors:** Olena Zimba, Burhan Fatih Kocyigit, Esha Kadam, Glenn Haugeberg, Simeon Grazio, Zofia Guła, Magdalena Strach, Mariusz Korkosz

**Affiliations:** 1 Department of Rheumatology, Immunology and Internal Medicine, University Hospital in Kraków, Kraków, Poland; 2 National Institute of Geriatrics, Rheumatology and Rehabilitation, Warsaw, Poland; 3 Department of Internal Medicine N2, Danylo Halytsky Lviv National Medical University, Lviv, Ukraine; 4 Department of Physical Medicine and Rehabilitation, University of Health Sciences, Adana City Research and Training Hospital, Adana, Türkiye; 5 Seth Gordhandhas Sunderdas Medical College and King Edwards Memorial Hospital, Mumbai, Maharashtra India; 6 Division of Rheumatology, Department of Internal Medicine, Sørlandet Hospital, Kristiansand, Norway; 7 Department of Neuromedicine and Movement Science, Faculty of Medicine and Health Sciences, NTNU, Norwegian University of Science and Technology, Trondheim, Norway; 8 Sestre milosrdnice University Hospital Centre, Zagreb, Croatia; 9 Department of Rheumatology, Physical and Rehabilitation Medicine, School of Medicine, University of Zagreb, Zagreb, Croatia; 10 Referral Centre for Spondyloarthritides, Ministry of Health of Republic of Croatia, Zagreb, Croatia; 11 UEMS PRM Board Training Centre in Physical and Rehabilitation Medicine, Zagreb, Croatia; 12 Department of Rheumatology and Immunology, Jagiellonian University Medical College, Kraków, Poland

**Keywords:** Axial spondyloarthritis, Diagnosis, Exercise, Rehabilitation, Surveys and questionnaires, Treatment

## Abstract

Spondyloarthritis (SpA) is a group of inflammatory disorders, including axial SpA (axSpA), characterized by inflammation in the spine and sacroiliac joints. Healthcare professionals have a crucial role in diagnosing and managing axSpA. Assessing their knowledge, perceptions, and practices is essential to enhance patient care. The objective of this study is to evaluate these factors by conducting an online survey. This online survey was performed using SurveyMonkey.com to assess healthcare professionals’ knowledge, perceptions, and practices related to axSpA diagnosis, management, and monitoring. The questionnaire included questions about definitions, management strategies, monitoring approaches, treatment options, and barriers to care. Convenience sampling was used, and the data were analyzed descriptively by Microsoft Excel. One hundred sixty-four healthcare professionals participated; most respondents were rheumatologists from various geographic locations (27 countries). Most participants were familiar with axSpA definitions and diagnostic criteria, demonstrating high expertise. Variations were seen in follow-up intervals and diagnostic preferences, reflecting clinical heterogeneity. Seventy-two (43.9%) individuals had a multidisciplinary team, frequently including rheumatologists, physiotherapists, and radiologists. Of the participants, 73 (44.5%) had online/telephone follow-up sessions. The pharmacological and non-pharmacological treatment approaches varied, pointing to the importance of personalized care. Glucocorticoid use varied among countries. Recognizing inflammatory back pain, interpreting radiographs, and diagnosing early was essential to medical education. This study provides beneficial data on healthcare professionals’ knowledge, perceptions, and practices regarding axSpA. While diagnostic familiarity and multidisciplinary approach are positives, there is a potential to standardize management, improve telemedicine services, remove barriers to physical activity, and optimize treatment options.

**Supplementary Information:**

The online version contains supplementary material available at 10.1007/s00296-024-05638-w.

## Introduction

Spondyloarthritis (SpA) is an umbrella term that describes a group of inflammatory diseases with comparable clinical manifestations and inherited features. Although there can be variations in reported data, the estimated prevalence in the general population is approximately 1% [1, 2]. The form of this group, termed axial SpA (axSpA), is distinguished by the prominent involvement of the spine and sacroiliac joints. Inflammatory back pain, stiffness, sleep disorders, and tiredness are characteristic for axSpA. AxSpA has two forms: radiographic axSpA (r-axSpA) and non-radiographic axSpA (nr-axSpA) [3, 4].

AxSpA has a diverse array of clinical signs. Regrettably, none of the individual attributes obtained from medical documentation, physical tests, laboratory outcomes, or radiologic screenings possess the precision required to identify axSpA conclusively. To diagnose axSpA, it is necessary to define a set of distinct patterns that, when considered together, offer sufficient evidence to confirm the presence of the disease [5]. Currently, numerous options are available to monitor axSpA. Most of the options depend on laboratory analyses, imaging assessments, and patient-reported outcomes [6, 7]. The treatment of axSpA involves a combination of non-pharmacological and pharmacological interventions. An individualized strategy is of utmost importance. It is crucial to combine non-pharmacological and pharmacological methods to treat axSpA [8, 9].

Healthcare professionals play a crucial role in diagnosing, managing, and monitoring patients with axSpA [10]. Gaining an insight into healthcare professionals’ knowledge, perceptions, and practices related to the diagnosis, management, and monitoring of axSpA is essential for enhancing the quality of care and optimizing outcomes.

This article presents findings from an online survey to assess health professionals’ knowledge, perceptions, and practices related to axSpA. By exploring health professionals’ understanding of axSpA diagnostic criteria, treatment modalities, and monitoring strategies, this study aims to identify unmet needs and areas for improvement in clinical practice.

## Methods

This survey aimed to assess healthcare professionals’ knowledge, perceptions, and practices regarding the diagnosis, management, and monitoring of axSpA patients. It was conducted using SurveyMonkey.com, an internet platform.

### Survey design

The survey questionnaire (Appendix 1) was designed based on an extensive review of current literature and EULAR practice guidelines [11, 12] to gather information on definitions, management strategies, monitoring approaches, and practices related to pharmacologic and non-pharmacologic treatments in axSpA. The questionnaire also reflected the views of healthcare professionals on obstacles to patients’ physical activities, strategies for reducing cardiovascular risk, the current status of online consultations, and the availability of multidisciplinary teams.

Five axSpA experts reviewed the questionnaire over two rounds of revisions to correct the questions, refine the wording, and ensure the consistency and validity of the content. This was followed by a simulated online form completion to evaluate the questionnaire in real time. To gather data, ten independent health professionals from different disciplines were requested to complete the survey. The survey outcomes were assessed, and feedback was obtained. Following this procedure, the questionnaire was revised and finalized. The final version of the questionnaire consisted of 33 questions, with 9 being multiple-choice, 17 single-answer, and one open-ended question. There were 6 sociodemographic questions.

Respondents could modify their responses before submitting them but not after the submission. All questions have been designated mandatory to ensure that the SurveyMonkey platform automatically removes incomplete responses.

### Sampling strategy

We employed a convenience sampling approach. The survey link was disseminated on X (Twitter) and Facebook from April 4 to 23, 2024.

### Ethics approval

The survey’s research protocol was approved by the Institutional Review Board (IRB) of the Jagiellonian University Medical College (protocol N 118.6120.07.2023, June 15, 2023). All participants provided informed consent before completing the questionnaire, with the assurance that their responses would be used solely for research purposes.

### Confidentiality, integrity, and availability

The survey used anonymization with Internet Protocol (IP) identities and participant emails as the only identifiable indicators. These indicators played an essential role in ensuring that each entry was unique to the individual. Data management ensured optimal anonymity as the authors stored IP addresses and emails only. Subsequently,  access to synthesized data displayed in tables without recognizable interaction was offered. We adhered to the latest recommendations on designing, planning, and reporting survey studies [13].

### Statistical analysis

The results section predominantly provided descriptive statistics. The normality of the distribution of all parameters was checked by the Shapiro-Wilk test. The descriptive statistics were reported using the following indicators: number (n), percentage (%), and median (minimum-maximum). Microsoft Excel was utilized to generate figures during the visualization process. Chi-square tests were employed to compare responses between groups. The results were deemed statistically significant at a P value of less than 0.05. The statistical analysis was conducted with Microsoft Excel.

## Results

### Baseline characteristics of participants

**Fig. 1:**
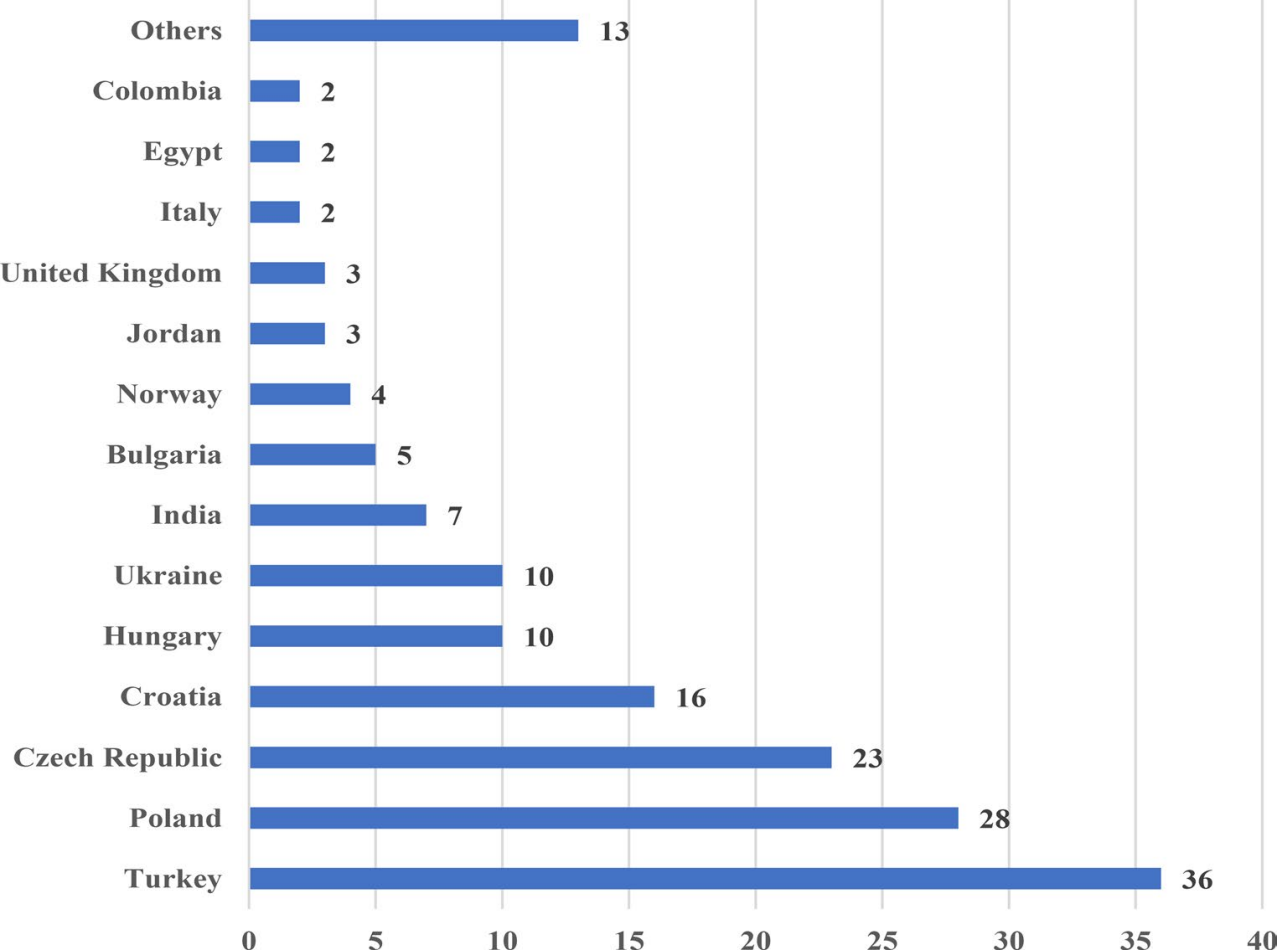
Country-wise distribution of respondents

A total of 164 individuals participated in the survey, with a median age of 42 (19–75) years. Out of the total participants, 85 (51.8%) were female, 75 (45.7%) were male, and 4 (2.5%) chose not to disclose their gender. The median duration following graduation was 17 (1–50) years. There were participants from 27 countries (Fig. 1). There were 129 consultant rheumatologists, 16 residents, 18 physiatrists, 2 general practitioners, and 9 individuals from allied professions. A total of 115 individuals were employed at the university teaching hospital, while 26 were employed at the outpatient center, 23 at the tertiary referral center, 7 were at the rehabilitation center, and 12 at other facilities. Additionally, 27 participants were employed at private practice.

### Knowledge about definitions and participant experience

The National Library of Medicine’s Medical Subject Headings (MeSH) introduced the definition of axSpA in 2022, and 151(92.1%) respondents were familiar with it. When assessing the patients in view of the Assessment in SpondyloArthritis International Society (ASAS) classification criteria for axSpA, 150 (91.5%) respondents used axSpA, nr-axSpA, or r-axSpA diagnostic terms. One hundred and forty-nine (90.9%) respondents were familiar with 2016 and 2022 updates of the ASAS-EULAR management recommendations for axSpA, with 8 (4.9%) responding ‘not sure’ and 7 (4.2%) responding ‘no’. A total of 97 (59.1%) respondents reported a special interest in axSpA, and 58 (35.4%) reported being a member of a dedicated axSpA clinic.

### Management strategies

Ninety-nine (60.4%) participants reported assessing axSpA patients at 3-month follow-up visits, 54 (32.9%) at 6-month, 3 (1.8%) at 9-month, and 8 (4.9%) at 12-month follow-ups. A total of 128 (78.1%) participants stated that individuals with axSpA typically seek care from either the general rheumatology department or the axSpA outpatient clinic when they experience flares. Meanwhile, 14 (8.5%) participants were unsure, and 22 (13.4%) responded no. When a patient with suspected axSpA is first examined, the preferences for imaging tests for the sacroiliac joints to confirm the diagnosis and/or fulfill the ASAS classification criteria were as follows: 92 (56.8%) responders employed both magnetic resonance imaging (MRI) and X-ray, 20 (13.5%) MRI only, and 50 (30.5%) X-ray only. When X-ray examination of the sacroiliac joints in patients with suspected axSpA was normal/uninformative, the choices of testing were as follows: 39 (23.8%) respondents used both sacroiliac joint and spinal MRI and 123 (75%) sacroiliac joint MRI only. Seventy-two (43.9%) participants had a multidisciplinary team/clinic managing axSpA patients at their centers. Of the participants working with a multidisciplinary team, 60 reported that rheumatologists, 31 rheumatology specialist nurses, 51 physiotherapists, 12 occupational therapists, 23 cardiologists, 14 clinical psychologists, and 47 musculoskeletal radiologists were members of the team.

**Fig. 2:**
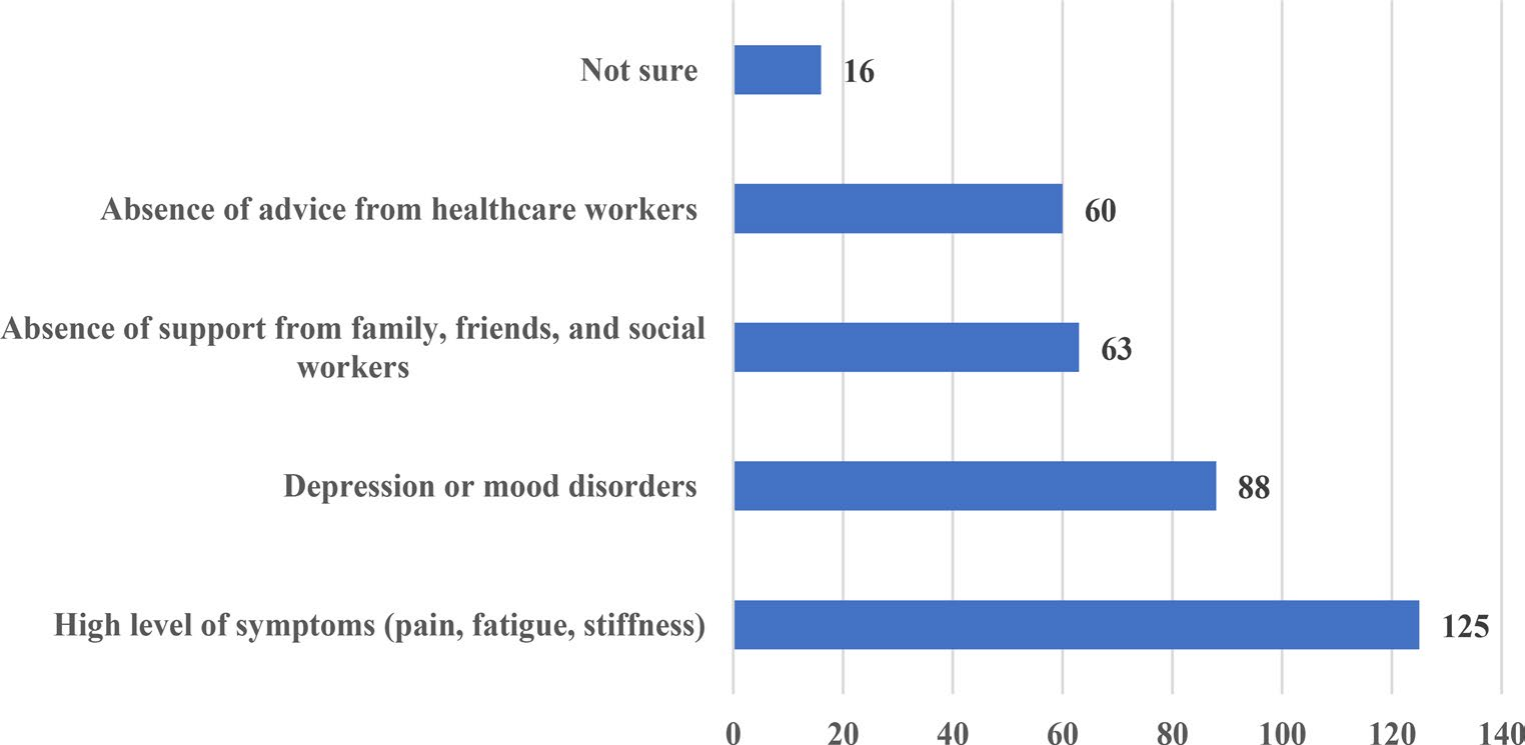
The main barriers to maintaining physical activity in patients with axSpA

Out of the all participants, 73 (44.5%) were engaged in online/telephone follow-up consultations to monitor the health and treatment compliance of axSpA patients. The three countries with the highest number of respondents were compared in terms of using online/telephone follow-up consultations. Although Polish responders frequently relied on such consultations, there were no statistically significant differences among the three countries (22.2% for Türkiye, 42.9% for Poland, and 21.7% for the Czech Republic; *p* = 0.134). The axSpA activity and quality of life measures used in the assessment process were reported as follows: 142 (86.6%) participants used Bath Ankylosing Spondylitis Disease Activity Index (BASDAI), 107 (65.2%) Ankylosing Spondylitis Disease Activity Score (ASDAS), 78 (47.6%) Bath Ankylosing Spondylitis Functional Index (BASFI), 42 (25.6%) Bath Ankylosing Spondylitis Metrology Index (BASMI), 18 (10.9%) Ankylosing Spondylitis Quality of Life Questionnaire (ASQOL), and 8 (4.9%) Work Productivity and Activity Impairment Questionnaire (WPAI). In addition, 16 (9.8%) participants reported using all scales. Six (3.7%) participants reported measuring the physical activity of axSpA patients using accelerometers. Four of these six participants were consultant rheumatologists, one was a physiatrist, and one was a resident. The factors seen as barriers to maintaining the recommended physical activity for patients with axSpA were as follows: high level of symptoms (pain, fatigue, stiffness) reported by 125 (76.2%) participants, depression or mood disorders by 88 (53.7%), absence of support from family, friends, and social workers by 63 (38.4%), and absence of advice from healthcare workers by 60 (36.6%) (Fig. 2). In axSpA, cardiovascular risk assessment priorities were the following: antihypertensive strategy was highlighted by 36, body weight control strategy by 40, lipid-lowering strategy by 34, smoking cessation strategy by 44, and all the mentioned by 147 participants. The number of participants who used the ASAS Health Index (ASAS HI) in their daily examination practice was 36 (21.9%); 99 (60.4%) did not use it; and 29 (17.7%) had no knowledge of this index.

The axSpA patients who should be treated by nonsteroidal anti-inflammatory drugs (NSAIDs) were reported as follows: 133 (81.1%) respondents mentioned about patients with pain and stiffness, 60 (36.6%) -  patients tolerating low-medium doses of NSAIDs, 119 (72.6%) -  symptomatic patients with active inflammation who tolerate maximal doses, and 101 (61.6%) -  patients without NSAIDs side effects.

**Fig. 3:**
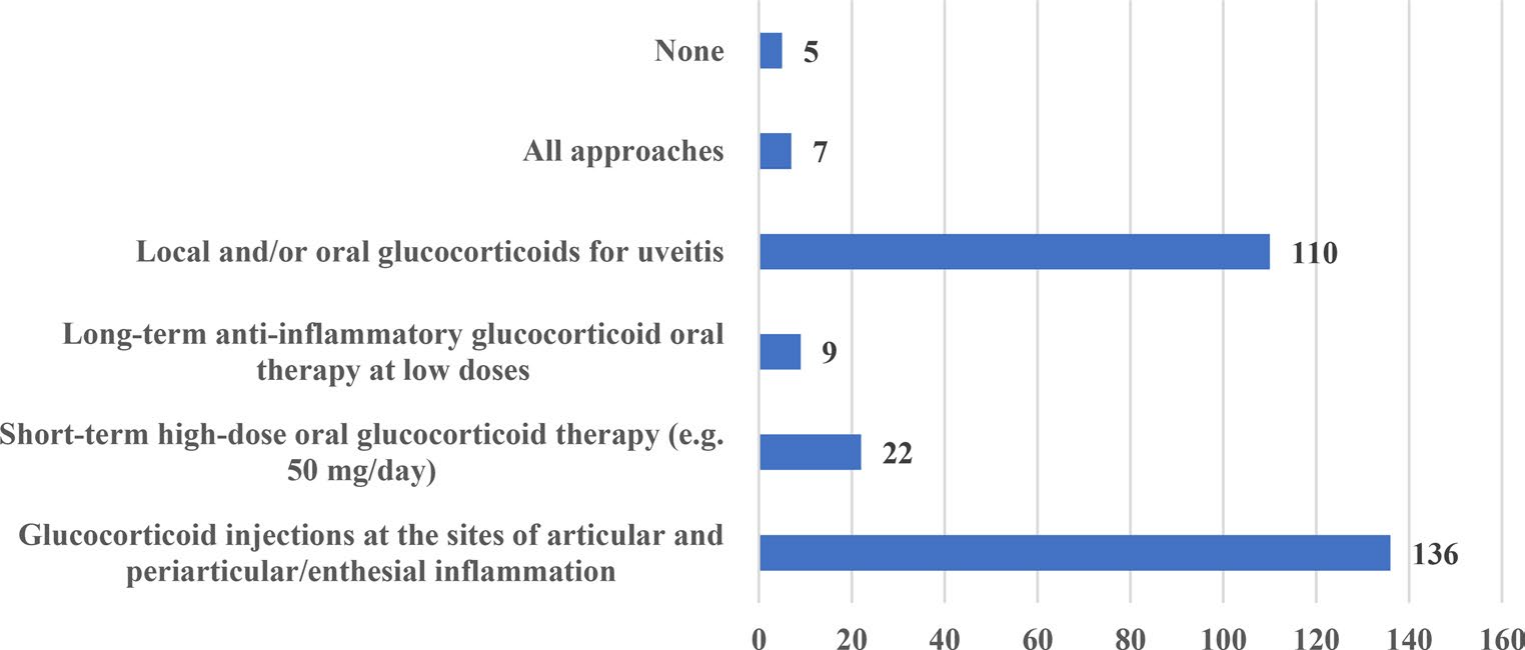
AxSpA glucocorticoid treatment strategies acceptable for the survey participants

The glucocorticoid treatment strategies acceptable for the participants were as follows: glucocorticoid injections at the sites of articular and periarticular/enthesial inflammation (136 [82.9%] participants), short-term high-dose oral therapy (e.g., 50 mg/day) (22 [13.4%]), long-term low-dose oral therapy (9 [5.5%]), local and/or oral therapy for uveitis (110 [67.1%]), all approaches (7 [4.3%]), and none (5 [3.1%]) (Fig. 3). The top three countries were selected based on the number of participants (Türkiye, Poland, and Czech Republic); steroid use strategies among these three countries were compared with the Chi-square test. There was no statistically significant difference in the use of glucocorticoid injections at the sites of articular and periarticular/enthesial inflammation, short-term high-dose oral glucocorticoid therapy, and long-term anti-inflammatory glucocorticoid oral therapy at low doses (*p* > 0.05). There was only a significant difference between countries in steroid use for the management of uveitis (*p* = 0.02) (92.8% for Poland, 69.6% for the Czech Republic, and 50% for Türkiye). The prominent countries in terms of the use of short-term high-dose oral glucocorticoid therapy were Türkiye (*n* = 6), Poland (*n* = 4), Ukraine (*n* = 3), and the Czech Republic (*n* = 3). The prominent countries in terms of the use of long-term anti-inflammatory glucocorticoid oral therapy at low doses were Türkiye (*n* = 2), Poland (*n* = 2), and Croatia (*n* = 2).

The conventional synthetic disease-modifying antirheumatic drugs (csDMARDs) of choice for peripheral manifestations/arthritis of axSpA were methotrexate (103 [62.8%] participants), sulfasalazine (143 [87.2%]), and leflunomide (29 [17.7%]).

**Fig. 4:**
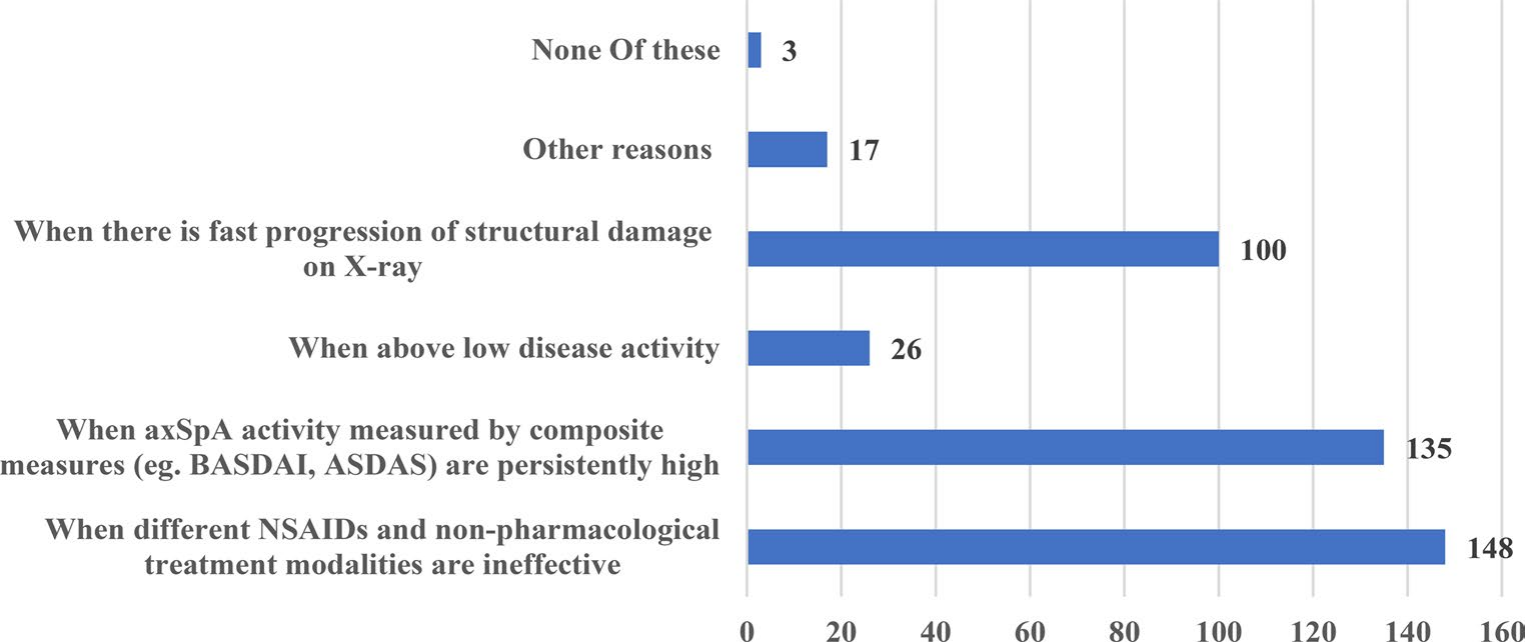
The main scenarios when biological drugs for axSpA are preferred the survey respondents

The scenarios where biologics such as tumor necrosis factor (TNF)-alpha inhibitors were preferred were as follows: 148 (90.2%) participants pointed to cases when different NSAIDs and non-pharmacological treatment modalities were ineffective, 135 (82.3%)  - when axSpA activity measured by composite measures (e.g. BASDAI, ASDAS) was persistently high, 26 (15.9%) -  when above low disease activity, and 100 (60.9%) -  when fast progression of structural damage on X-ray. (Fig. 4).

When TNF-alpha inhibitor therapy failed to suppress inflammation (secondary ineffectiveness, not side effects), the preferred treatment strategies were as follows: 44 (26.8%) participants reported administering another TNF-alpha inhibitor, 64 (39%) -  administering anti-IL-17 therapy, 32 (19.5%) -  administering a JAK inhibitor, and 91 (55.5%) reported that all three options were applicable. The number of participants who routinely applied non-pharmacological treatment modalities was 143 (87.2%). The number of participants who discussed the most preferred treatment modalities and best possible management plans with their patients with axSpA and/or their caregivers as part of a shared decision-making process was 146 (89%). The number of participants who considered costs incurred when evaluating the cost-effectiveness of imaging/treatment modalities, particularly biologic/targeted synthetic drugs, was 126 (76.8%).

The number of participants who encountered patients who developed axSpA after recovering from COVID-19 was 57 (34.8%).

An open-ended question was used to identify priorities in specialty training for diagnosis and management of patients with axSpA. The following three main themes were mentioned by the participants: assessment of inflammatory back pain (*n* = 40), radiologic examination (*n* = 37), and early diagnosis (*n* = 28).

## Discussion

This survey’s results provide insights into healthcare professionals’ knowledge, perceptions, and practices regarding the diagnosis, management, and monitoring of axSpA. Gaining a comprehensive understanding of these factors is crucial for enhancing the quality of patient care and achieving favorable outcomes in the management of axSpA.

The majority of the survey participants were consultant rheumatologists. The median length of experience following graduation was seven years. More than half of the participants were employed at the university teaching hospital. The countries with the highest number of respondents were Turkiye (*n* = 36), Poland (*n* = 28), and Czech Republic (*n* = 23). It is anticipated that rheumatologists will mostly participate in a survey related to axSpA, given this is one of their primary focus areas [14].

AxSpA predominantly affects the axial skeleton and sacroiliac joints. Currently, ASAS-EULAR recommendations exist for the management of patients [12, 15]. Most respondents were acquainted with the definition of axSpA. In addition, many participants demonstrated familiarity with the diagnostic terminology utilized in the ASAS classification criteria for axSpA. Moreover, a substantial number of respondents indicated their acquaintance with the ASAS-EULAR management recommendations, demonstrating their knowledge of the current standards for managing axSpA.

The survey uncovered variances in the frequency of follow-up visits for axSpA patients, with the majority choosing 3-month intervals. However, there were preferences for 6-month and 12-month follow-ups. This variability may be due to variances in patient populations, disease severity, and healthcare system characteristics.

Imaging of the sacroiliac joints is a crucial component for evaluating axSpA [16]. Participants preferred both MRI and X-ray investigations when diagnosing axSpA. However, there was also a broad adoption of MRI or X-ray alone. Diagnostic approaches differ, affecting the diagnostic accuracy and patient outcomes. Standardized protocols or guidelines can help to expedite diagnostic paths and optimize resource utilization [17, 18].

Approximately half of the participants reported engaging multidisciplinary teams in managing axSpA. This highlights the acknowledgment of the intricate nature of axSpA and the requirement for comprehensive care. Rheumatologists were the most commonly reported members of multidisciplinary teams, followed by physiotherapists and musculoskeletal radiologists. This interdisciplinary approach aligns with current recommendations and allows comprehensive care customized to meet each patient’s specific needs [19, 20].

Nearly 50% of participants reported utilizing online/phone follow-up consultations to monitor axSpA patients. This indicates the potential for extending telemedicine services in the management of axSpA. Telemedicine has an opportunity for remote monitoring, prompt intervention, and improved healthcare accessibility, particularly for patients residing in rural regions or with restricted mobility [21, 22].

Barriers to sustaining suggested physical activity for axSpA patients were primarily symptom-related, emphasizing the impact of pain, fatigue, and stiffness on physical function and quality of life. Addressing these obstacles with individualized exercise initiatives, psychological assistance, and education may increase compliance with physical activity recommendations and enhance patient outcomes.

The survey yielded valuable information regarding pharmacological treatment approaches for axSpA, encompassing the utilization of NSAIDs, glucocorticoids, csDMARDs, and biologic/targeted synthetic DMARDs. NSAIDs were often given for symptom alleviation. Glucocorticoid treatment approaches exhibited variability, with a preference for local injections, short-term oral therapies, and long-term low-dose therapies. These findings demonstrate the intricacy of controlling symptoms associated with axSpA and emphasize the necessity for personalized treatment strategies [23].

It was emphasized that biologic drugs, particularly TNF-alpha inhibitors, are preferred for individuals who do not respond to NSAIDs or have high disease activity. Furthermore, the survey looked into the secondary ineffectiveness of TNF-alpha inhibitors and other treatment options. Participants indicated that a different TNF-alpha inhibitor, IL-17 inhibitor, and JAK inhibitor would be appropriate.

Most respondents employed non-pharmacological management approaches, highlighting the need for holistic approaches to axSpA care. Decision-making collaboratively and cost-effectiveness considerations in assessing treatments were also frequently reported, indicating patient-focused and value-based concepts.

Similarities emerged when the top three countries’ glucocorticoid use strategies were compared. However, there was a difference in uveitis management approaches. It highlights the possible impact of geographical characteristics such as health systems, cultural practices, and resource availability on treatment decisions and outcomes.

The open-ended question unveiled critical areas of focus for medical specialty training in axSpA, encompassing the evaluation of inflammatory back pain, radiographic analysis, and early diagnosis. By addressing these training gaps, healthcare providers can improve their proficiency in detecting and managing axSpA, leading to better patient outcomes.

## Conclusion

This study offers helpful information about healthcare professionals’ knowledge, perceptions, and practices regarding axSpA. Although diagnostic familiarity and multidisciplinary care have advanced, there are also unmet needs in standardizing management approaches, enhancing telemedicine services, overcoming obstacles to physical activity, and optimizing pharmacological and non-pharmacological treatment strategies. Additionally, continuous education and training designed to meet country-based requirements are crucial for improving axSpA treatment and enhancing patient outcomes.

### Electronic supplementary material

Below is the link to the electronic supplementary material.

Supplementary Material 1
