# Supplementary material for: Knowledge, perceptions, and practices of axial spondyloarthritis diagnosis and management among healthcare professionals: an online cross-sectional survey
Source: Rheumatol Int. 2024 Jun 22;44(8):1501–8. doi: 10.1007/s00296-024-05638-w (PMC11222259; doi:10.1007/s00296-024-05638-w)
Supplement: Supplementary file 1 — Supplementary Material 1 [file 296_2024_5638_MOESM1_ESM.pdf]

Knowledge, perceptions and practices related to diagnosis and management of axial spondyloarthritis

The current survey is aimed to examine specialists' knowledge, perceptions, and practices related to axial spondyloarthritis (axSpA) diagnosis and management. This questionnaire is designed based on previous similar questionnaires, relevant global practice guidelines (i.e., *2016 and 2022 updates of the ASAS-EULAR management recommendations for axial spondyloarthritis*), and reviews.

The respondents are expected to completely fill this questionnaire and share their knowledge and experience as clinicians and specialists undergoing life-long education and diagnosing and managing patients with axSpA. By completing this survey questionnaires, respondents give their agreement to publicize their answers. Although there are some personal demographic and clinical experience questions about responders, all answers will be kept confidential and anonymised. Only questionnaires with complete answers to all questions will be processed.

Thank You for your professional cooperation on this matter!

For further questions, Associate Professor Olena Zimba can be contacted at zimbaolena@gmail.com

Knowledge, perceptions and practices related to diagnosis and management of axial spondyloarthritis

* 1. Are you familiar with the following definition of axial spondyloarthritis (axSpA) which was introduced in 2022 by the Medical Subject Headings (MeSH) of the National Library of Medicine of US (https://www.ncbi.nlm.nih.gov/mesh/2101123): "A spectrum of chronic inflammatory conditions affecting the axial joints (e.g., SPINE), characterized by pain, stiffness of joints (ANKYLOSIS), reduced mobility and inflammation. When joint inflammation and damage are visible on regular X-rays it is called ANKYLOSING SPONDYLITIS; otherwise, it is referred to as NON-RADIOGRAPHIC AXIAL SPONDYLOARTHRITIS".

- ◯ Yes
- ◯ No
- ◯ Not sure

* 2. Do you label the patient with diagnostic terms of axial spondyloarthritis (axSpA) or Non-radiographic axSpA(nr-axSpA) or radiographic axSpA (r-axSpA, ie. ankylosing spondylitis) when assessing the patient and using ASAS classification criteria for axSpA (https://pubmed.ncbi.nlm.nih.gov/19297344/)?

- ◯ Yes
- ◯ No

* 3. Are you familiar with 2016 and 2022 updates of the ASAS-EULAR management recommendations for axSpA?

- ◯ Yes
- ◯ No
- ◯ Not sure

* 4. Do you have a special interest in axSpA?

- ◯ Yes
- ◯ No
- ◯ Not sure

* 5. Are you a member of a dedicated axSpA clinic in your centre?

- ◯ Yes
- ◯ No
- ◯ Not sure

* 6. How often do you assess axSpA patients on follow-up visits?

- ◯ Every 3 months
- ◯ Every 6 months
- ◯ Every 9 months
- ◯ Every 12 months

* 7. Can your axSpA patients self-refer to general rheumatology department or axSpA out-patient clinic when they experience flare-ups?

- ◯ Yes
- ◯ No
- ◯ Not sure

* 8. When you first time examine a patient with suspected axSpA, which of the following imaging tests for sacroiliac joints (SIJ) are ordered in your centre to confirm the diagnosis and/or fulfilment of ASAS classification criteria?

- ◯ SIJ X-ray
- ◯ SIJ MRI
- ◯ Both SIJ MRI and X-ray
- ◯ None

* 9. When X-ray exam of sacroiliac joints (SIJ) in patients with suspected axSpA is normal/not informative, which tests you would prefer to order next?

- ◯ MRI of SIJ
- ◯ MRI of the whole spine
- ◯ MRI of both whole spine and SIJ
- ◯ None of these

* 10. AxSpA often requires multidisciplinary management to assess and manage peripheral and extra-articular manifestations along with comorbidities (eg. cardiovascular). Do you have a multidisciplinary team/clinic in your centre managing axSpA patients?

- ◯ Yes
- ◯ No

Knowledge, perceptions and practices related to diagnosis and management of axial spondyloarthritis

* 11. If you have a multidisciplinary axSpA team/clinic, who are members of this team? Select all that apply

- ☐ Rheumatologist
- ☐ Rheumatology specialist nurse
- ☐ Physiotherapist
- ☐ Occupational therapist
- ☐ Cardiologist
- ☐ Clinical psychologist
- ☐ Musculoskeletal radiologist
- ☐ No, we do not have a multidisciplinary team/clinic

* 12. Do you run online/phone follow-up consultations for axSpA patients to monitor their health and treatment adherence?

- ○ Yes
- ○ No

* 13. Which axSpA activity and quality of life measures are calculated/processed in your practice? Select all that apply.

- ☐ Bath Ankylosing Spondylitis Disease Activity Index (BASDAI)
- ☐ Ankylosing Spondylitis Disease Activity Score (ASDAS)
- ☐ Bath Ankylosing Spondylitis Functional Index (BASFI)
- ☐ Bath Ankylosing Spondylitis Metrology Index (BASMI)
- ☐ Ankylosing Spondylitis Quality of Life Questionnaire (ASQOL)
- ☐ Work Productivity and Activity Impairment Questionnaire (WPAI)
- ☐ All of the above
- ☐ None of the above

* 14. Do you measure your axSpA patients’ physical activity using an accelerometer (e.g., Actigraph wGT3X-BT)?

- ○ Yes
- ○ No
- ○ Not sure

* 15. Which of the following factors are viewed as barriers to recommended maintenance of physical activity of your patients with axSpA? Select all that apply

- ☐ High level of symptoms (pain, fatigue, stiffness)
- ☐ Depression or mood disorders
- ☐ Absence of support from family, friends, and social workers
- ☐ Absence of advice from healthcare workers
- ☐ Not sure

* 16. Cardiovascular risk assessment in axSpA should be focused on the following: Select all that apply.

- ☐ Antihypertensive strategy
- ☐ Body weight control strategy
- ☐ Lipid-lowering strategy
- ☐ Smoking cessation strategy
- ☐ All of the above
- ☐ None of the above

* 17. The primary long-term goal of managing patients with axSpA is to maximise health-related quality of life and health status through control of inflammation, prevention of progressive structural damage, preservation of function and social participation. In this regard, the Assessment of SpondyloArthritis International Society Health Index ((ASAS HI; based on the International Classification of Functioning, Disability and Health (ICF) https://www.asas-group.org/instruments/asas-health-index/: contains items addressing categories of pain, emotional functions, sleep, sexual function, mobility, self-care, and community life) and was recommended by ASAS-EULAR to monitor patients’ status. Do you use ASAS HI in your practice? your practice?

- ○ Yes
- ○ No
- ○ Unaware of this index

* 18. Which axSpA patients should be administered NSAIDs? Select all that apply.

- ☐ Patients with pain and stiffness
- ☐ Patients tolerating low-medium doses of NSAIDs
- ☐ Symptomatic patients with active inflammation who tolerate maximal doses
- ☐ Patients without NSAIDs side effects

* 19. Which of the following glucocorticoid treatment strategies you would approve for axSpA? Select all that apply.

- [ ] Glucocorticoid injections at the sites of articular and periarticular/enthesial inflammation
- [ ] Short-term high-dose oral glucocorticoid therapy (e.g. 50 mg/day)
- [ ] Long-term anti-inflammatory glucocorticoid oral therapy at low doses
- [ ] Local and/or oral glucocorticoids for uveitis
- [ ] All of these
- [ ] None of these

* 20. Which of the following conventional synthetic DMARDs(csDMARDs) therapies you would prefer for peripheral signs/arthritis of axSpA? Select all that apply.

- [ ] Methotrexate
- [ ] Sulfasalazine
- [ ] Leflunomide
- [ ] None of these

Knowledge, perceptions and practices related to diagnosis and management of axial spondyloarthritis

* 21. When would you prefer to use biologics such as anti-TNF-alpha inhibitors? Select all that apply.

- [ ] When different NSAIDs and non-pharmacological treatment modalities are ineffective
- [ ] When axSpA activity measured by composite measures (eg. BASDAI, ASDAS) are persistently high
- [ ] Above low disease activity
- [ ] When there is fast progression of structural damage on X-ray
- [ ] Other conditions-please name

* 22. Your preferred strategy when anti-TNF-alpha inhibitor therapy fails (secondary inefficacy, not side effects) to suppress inflammation? Select all that apply.

- [ ] Administer another anti-TNF-alpha inhibitor
- [ ] Administer anti-IL-17 therapy
- [ ] Administer JAK inhibitor
- [ ] All of three above could apply
- [ ] None of these

* 23. Do you routinely administer non-pharmacological treatment modalities for your patients with axSpA?

- ( ) Yes
- ( ) No

* 24. Do you discuss with your patients with axSpA and/or their caregivers the most preferred treatment modalities and the best possible management plans as part of shared decision-making?

- ( ) Yes
- ( ) No

* 25. AxSpA incurs high financial costs for patients and society. Do you consider costs incurred when evaluate cost-effectiveness of imaging/treatment modalities, and particularly with biologic/targeted synthetic drugs?

- ( ) Yes
- ( ) No

* 26. Have you encountered subjects who developed axSpA after recovering from COVID-19?

- ◯ Yes
- ◯ No

* 27. Which topics should be prioritized in medical specialty education to timely diagnose and manage patients with axSpA?

Knowledge, perceptions and practices related to diagnosis and management of axial spondyloarthritis

Demographics

* 28. Age

18 years — 80 years

* 29. Gender

- Male
- Female
- Prefer not to answer

* 30. Country of current medical practice

* 31. Years in medical practice post graduation of medical school/university

1 year — 40 years

* 32. What is your specialty?

- Consultant rheumatologist
- Resident
- Specialist nurse
- Physiotherapist
- Physiatrist
- General Practitioner
- Other (please specify)

* 33. Where are you employed?

- [ ] University teaching hospital
- [ ] Outpatient centre
- [ ] Tertiary referral centre
- [ ] Rehabilitation centre
- [ ] Private practice
- [ ] Other (please specify)
